# Smart Software Can Increase Sit–Stand Desk Transitions During Active Computer Use

**DOI:** 10.3390/ijerph16132438

**Published:** 2019-07-09

**Authors:** Pankaj Sharma, Adam Pickens, Ranjana Mehta, Gang Han, Mark E. Benden

**Affiliations:** 1 Department of Environmental & Occupational Health, Texas A&M University School of Public Health, College Station, TX 77843, USA; 2 Department of Biostatistics & Epidemiology, Texas A&M University School of Public Health, College Station, TX 77843, USA

**Keywords:** software, office physical activity, sedentary behavior

## Abstract

The objective use of table top adjustable sit–stand desks has yet to be determined, due to the lack of an effective digital evaluation method. The objective of this study was to evaluate the impact of computer prompt software on table top sit–stand desks to determine if there was a difference in the frequency of desk position changes. This five month, pre-post pilot study on 47 university staff members used a novel USB accelerometer sensor and computer software reminders to continuously record and prompt increases in desk usage to promote physical activity at the workstation. During the baseline phase (3 months), desk usage data were continuously recorded for all workers. Following the baseline, the results from a two-month intervention of personalized computer reminders doubled the number of desk position changes per work day from 1 desk position change every 2 work days to 1 change every work day. Furthermore, those who changed desk positions once or twice a day increased from 4% to 36% from baseline to intervention. Overall, the intervention was encouraging, but longer intervention studies are warranted to determine if the desk usage behavior change can be improved and sustained for years and whether that change results in health gains.

## 1. Introduction

The increase in technological developments in buildings have allowed humans to expend less energy, and this has contributed to the workplace obesity epidemic [1]. Traditional office buildings have been designed in the direction of “human energy conservation”, including conveniently located elevators along with many sitting options [1]. Given the evidence of the effects of prolonged sitting on health (cardiovascular disease, type 2 diabetes, venous thrombosis), experiments have shown that breaks from prolonged sitting every 30 min can elicit benefits to cardiometabolic health [2,3]. Standing for too long can also be detrimental to health. Prolonged standing periods have been associated with lower back discomfort [4], carotid atherosclerosis [5], varicose veins [6], and muscle fatigue [7]. As a result, activity-permissive workstations allow people to change postures between sitting and standing and are being incorporated into the workplace to reduce workers’ sedentary behavior and improve associated health outcomes [8].

On the market today, the two primary types of sit–stand workstations are stand-biased and height-adjustable (electric, crank, pneumatic, and now trending, table top adjustable). Due to its ready-to-use design out of the box, most table-top adjustable desks save assembly time, and provide ergonomic adjustability. The desks require worker action (lift or lower the desk) to change to sitting and standing heights.

While the need to increase transitions has been established by the 2015 study by Hedge et al. with a generic guidance of 20/8/2 (20 min seated then 8 min standing and then 2 min walking per half hour), no studies have evaluated objectively whether table top unit sit–stand users are close to the recommendation [9]. In our other studies using software to monitor electric sit–stand table use, we found 8–15% of the shift being used in a standing position with on average only 1 desk transition from a sit to a stand position per shift. If table top units follow similar patterns (they were less frequent per our current study), then they would benefit from some form of behavioral reminder. Since we have had success making improvements to transitions in other studies, we chose to attempt to do the same with the table top units [9,10,11].

Other studies have used software reminders to reduce sitting time and increase position change at work [10,11,12]. However, they have been short-term studies and did not account for the time that workers spend away from their office workstation, due to other work activities that take place away from the desk during the day. One recent longitudinal study used software reminders to prompt and increase workers’ electric sit–stand desk usage, while taking into account time spent away from the workstation [13]. Mechanical sit–stand desk units, to date, have only been studied by subjective survey of user habits. As such, methods to objectively collect baseline and or intervention data on usage of these devices has not been available. The objective of this study was to determine the impact of personalized software reminders on mechanical table top adjustable sit–stand desk usage.

The objective of this study was to evaluate the impact of computer prompt software on table top sit–stand desks to determine if there was a difference in the frequency of desk position changes. A unique aspect of this study was that a validated USB accelerometer was mounted on the mechanical sit–stand desks as a novel method to collect continuous data on desk usage for the entire work day.

## 2. Materials and Methods

As per Texas A&M University IRB approved protocol, all data were de-identified by Texas A&M University Division of Student Affairs Information Technology and then provided to Texas A&M University School of Public Health researchers for secondary analyses.

### 2.1. Participants

Participants were staff members from the Texas A&M University Division of Student Affairs in College Station, Texas. Office work consisted of computer-based tasks and meetings with students. As part of a university initiative to promote an active workplace, mechanical table top sit–stand desks were provided to a group of 162 employees who provided a reason/justification. The staff were distributed among 12 buildings throughout the campus. Office designs were traditional, individual offices for each staff member. The Texas A&M University School of Public Health researchers and Division of Student Affairs leaders recruited workers for the study via e-mails. Workers were provided information with regards to the study design and length. The interested staff members responded through an online survey indicating their interest.

Out of 162 staff, 65 chose not to participate in the study and 97 expressed interest to participate. The 97 workers were provided the Texas A&M University Institutional Review Board approved consent form to participate in the study. Out of the 97 interested potential participants, 74 workers submitted a signed consent form. During the five-month data collection period, 74 staff members had recorded data. The inclusion criteria for this secondary analysis was ≥ 20 ACU (active computer use) hours and ≥ 5 work days per worker during each study phase (baseline & intervention), which was equivalent to one work week pre and post. Out the 74 staff, 47 workers were eligible for this analysis (n = 47).

### 2.2. Data Collection

**Figure 1:**
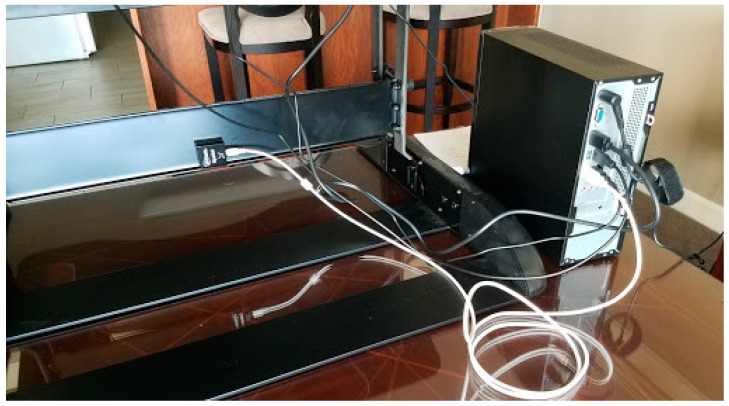
Setup of the mechanical table top desks.

Data were collected through a validated computer software (Wellnomics^®^ Sit-stand Texas A&M, Christchurch, New Zealand) which recorded the number of desk position changes and the time desks were in a sitting or standing position during active computer use. This software has been previously used to determine the same outcome measures for over 300 workers using electric sit–stand desks, and the methods on how the software collects data is explained in the study [14]. Since this software was used in conjunction with a non-electric sit–stand desk, a novel approach was used by integrating a lab-validated USB accelerometer sensor with the computer software (Figure 1). The sensor (Phidget Spatial 3-axis ±8g accelerometer) provided information to the software on the position of the desk and was validated in the lab by comparing video timestamps with raw data output from the computer software (+/− 2.6% agreement) for time periods of 5 h on 5 separate days. The primary outcome measure for this study was the frequency of desk position changes. Other measures included the time desks were in a sitting and standing position and the maximum time the desks were in a position before a change was made. Additionally, since sit–stand time was based on ACU time, we also looked at the proportions of sit vs. stand time as a percent of computer use, as it provides context for the perceived effectiveness of the mechanical desk. A short, non-validated questionnaire about co-worker influence on sit–stand desk usage was sent to all participants at the beginning of the study (Table 1). This instrument was created in conjunction with TAMU staff in an attempt to understand whether future interventions should be directed at peer influence strategies such as gamification where team scores for behavior might be displayed and communicated to create competition toward PA goals. Researchers were not provided with any demographic information (gender, age) on the workers.

### 2.3. Study Design: Baseline

The data were collected for a five-month period. The adjustable sit–stand desks were given to the workers during the Summer of 2016. The sensor was validated in the lab during the summer of 2017 and workers were recruited for the study during August of 2017. The validated USB accelerometer sensor was mounted on all participants’ desks by a Texas A&M researcher in September 2017, and the software was installed for all participants in October 2017 by the Division of Information Technology. After installation, the software required workers to complete an ergonomic setup for their table top adjustable sit–stand desk.

Once the setup was completed by each worker, the software informed each worker through a reminder that the software application was going into *monitor only* mode (recorded continuous data on desk sitting and standing time and worker continued work as normal). From 1 November 2017 through 31 January 2018, the software measured the primary outcome of the number of desk position changes completed by the staff members. Additionally, the software recorded the time desks were in a sitting or standing position and the maximum time the desks were in a position before a change was made by the worker (both during active computer use).

### 2.4. Study Design: Intervention

**Figure 2:**
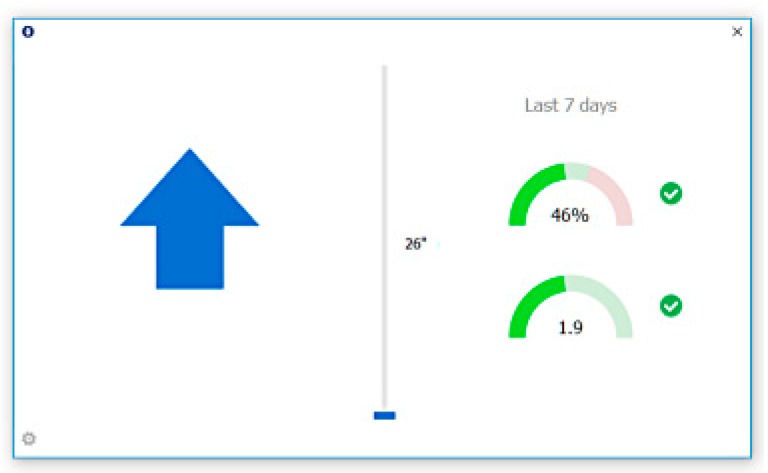
Screenshot of the software prompt where workers could interact with their personal statistics.

On 1 February 2018, all enrolled workers were sent an email describing the second phase of the study (intervention), which consisted of software reminders to change desk positions. From 1 February 2018 through 30 March 2018, the software was in *reminder* mode and recorded the aforementioned outcomes. The default setting for the reminders was 30 min sitting then a reminder to stand for 20 min [15,16,17,18]. Each worker could change the reminder settings to their preferences or ignore the reminders. Additionally, real-time statistics on personalized desk usage (number of desk position changes per work day & percent ACU time desk in a standing position) were provided when the workers opened the software application (Figure 2).

### 2.5. Analysis

The analysis was conducted using SPSS 22 (SPSS Inc., Chicago, IL, USA). Pairwise comparison analysis was conducted to test differences between baseline and the intervention using means, SD, and histograms. We tested for significance of the intervention using paired two sample for means t-test. The overall pooled means were calculated for every month of data collection period to determine usage patterns before and after the intervention. Two weeks of company holiday period were excluded from this analysis (25 December 2017–5 January 2018).

## 3. Results

For the baseline phase, data from a mean of 112 ACU hours and 34 work days per worker were recorded. For the intervention phase, data from a mean of 82 ACU hours and 31 work days per worker were recorded. Due to site specific issues resulting in low participation, 3 weeks of data was excluded from the secondary analysis (25 December 2017–5 January 2018 and 13–24 November 2017) for vacation and pilot respectively. Pairwise comparisons of difference in means for the outcome measure are shown in Table 2. Two outcome measures (desk position changes per work day and maximum ACU time desk in a position before a change) significantly improved from the intervention (*p* < 0.001). Stand or sit ACU did not significantly change from the intervention.

**Figure 3:**
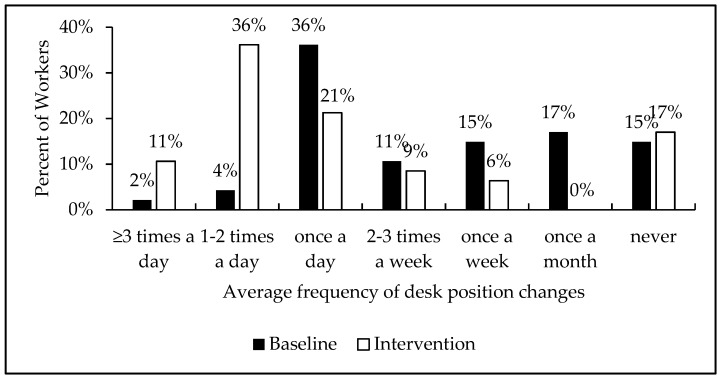
Frequency of desk position changes among workers. The average frequency of desk position changes as a percent of staff members from baseline to intervention are represented here.

Figure 3 shows the differences among all workers in the frequency of desk position changes per work day. The largest sub-population of workers with the greatest improvement were those in the 1–2 desk position changes per work day. Out of 47 staff members, these group of 2 staff members (4%) increased to 17 staff members (36%) after the intervention. Another important finding was the elimination of the sub-group of staff members who used to change desk positions once a month (8 staff members to 0 staff members), indicating that the software reminders were successful at getting the lowest frequency users to change position.

**Figure 4:**
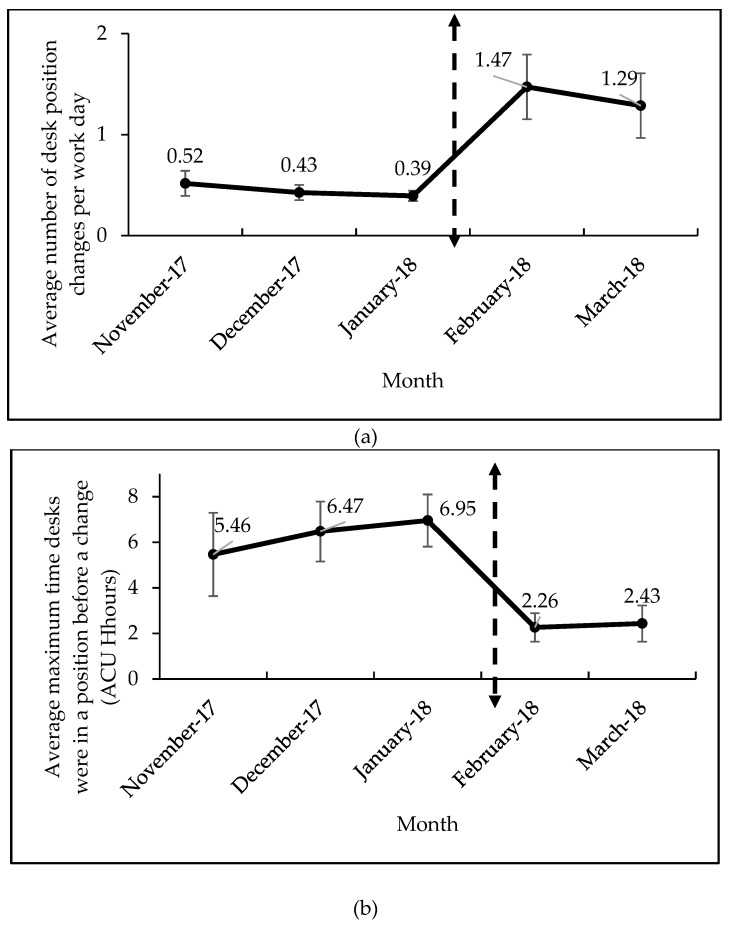
Overall group means of desk position changes and max time desk in a position during each month of the study. Desk position changes per work day (**a**) and average maximum time desks were in a position before a change in ACU hours (**b**) are shown. Dashed line with two arrows indicates the beginning of the intervention. 25 December 2017–5 January 2018 data were excluded from this analysis. Error bars represent 95% confidence intervals.

Figure 4 illustrates the monthly trends for two outcome measures (average number of desk position changes and maximum ACU time the desk was in a position) at each phase of the study: baseline (November 2017, December 2017, January 2018) and intervention (February & March 2018). For the average number of desk position changes (Figure 4a) and the maximum ACU time the desks were in a position before a change (Figure 4b), there was an overall improvement from the computer reminders from beginning to the end of the study. During the baseline phase, staff members completed 1 desk position change per 2 work days. After the intervention, they completed 1 desk position change per 1 work day.

**Figure 5:**
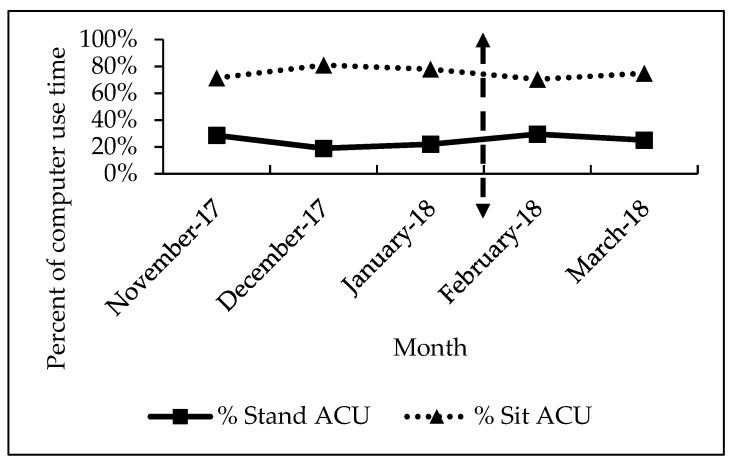
The time desks were in sitting and standing position as a percent of computer use. Dashed line with two arrows indicates the beginning of the intervention.

Figure 5 displays sit vs. stand ACU as a percent of computer use for each month of the study. During the baseline phase, the staff members had their desk in the sitting position 77% of computer use time and the desk was in a standing position for the remaining 23% of computer use time. After the intervention was complete, the staff members reduced sedentary time and had their desk in the sitting position 72% of computer use time, and the desk was in a standing position for the remaining 28% of computer use time. These changes from the intervention were not statistically significant.

The survey results revealed that 51% of the participants (24 out of 47 staff members) were influenced by their co-workers’ habits with their sit–stand desks and 49% were not influenced. Specifically, the staff members were influenced when either they discussed their sit–stand habits with their co-workers or when they saw them using their sit-stand desks. Out of the 51% who were influenced, all of them (100%) agreed that they were motivated to stand more and change desk positions more frequently because of their co-workers.

## 4. Discussion

The objective of this study was to evaluate the impact of computer prompt software on table top sit–stand desks to determine if there was a difference in the frequency of desk position changes. Overall, the intervention increased sit–stand desk usage for the staff members. From the software reminders, the number of desk position changes per work day showed a statistically significant change, as it doubled compared to the baseline. This is similar to effect seen in our other study with the use of electric sit–stand desks, showing that the software is effective in increasing desk position change frequency [13]. The intervention successfully broke up long bouts of the desk being in one position during computer use (5.5–7 h during the baseline to ~2.5 h during the intervention), which was also statistically significant. This reveals that the staff members were able to reduce the amount of time between desk position changes, which is an important implication for breaking up long periods in a sitting or standing position. It should be noted that this is still a long way from the goal of transitions every 30 min noted in the Hedge reference and clearly an opportunity for table top sit–stand designers to continue to improve their products utilization [9]. Although the stand–sit ACU time change from the intervention was not statistically significant, the workers still had more desk transitions during the work day and broke up long periods of time in which the desk was in one position. The software reminders successfully changed and improved the sit-stand desk behavior for the staff members.

The main strength was that all staff members had their sit–stand desk for at least 1 year prior to the start of the study. The desks were not given to participants for the software intervention, thereby eliminating any potential effect from the novelty of a new desk [19]. Another strength was the novel approach of measurement using the USB sensor and validated software for minute by minute digital monitoring of the desktop PC and desk height for 5 months. Most related studies to date have used worker observation or recall for very short durations such as a week or a day.

The main limitation of the study was the recruitment pool of participants for the study. All potential participants requested a sit–stand desk through the university and therefore were the only ones eligible to participate in the study. Furthermore, only the individuals who signed a consent form could be participants of the study. The individuals who signed a consent form could have primarily joined the study because they wanted to make a physical activity change, which could have created volunteerism bias. The introduction of a setup and prompt warning of the “*monitor only mode*” to follow could have influenced behavior over the next few months. Future software interventions should be at least 1 year in duration to determine if the behavior change is maintained and the pool of participants should include those randomly selected from locations where all workers use the sit–stand desks.

This study provided objective data for a population of mechanical sit–stand desk users. In general, the users of this style of desk spent more time standing and had less frequent desk position changes when compared to a similar study on electric sit–stand desk user habits [13]. For both studies, once software was added, the differences faded over time, showing the need for longer, sustainable interventions. As mentioned in the limitations, we focused on the time spent at the computer due to our measurement approach, but the other 16–20 h of the day should also be evaluated with wearables to evaluate for any compensatory behaviors when gains are made at work.

## 5. Conclusions

This is the first study to measure digital, objective data on table top adjustable sit–stand desk use. Results show potential for computer-based interventions of sedentary behavior given modest improvements and high compliance with prompts. The potential for mechanical desk top sit–stand units to perform at least as well as their electric desk comparators when evaluating time sitting and standing and position changes once the software is deployed was also evident. Most notable on the intervention side was that the people in need of movement the most responded the most to the software intervention. It is always positive when public health interventions applied across pilot populations yield benefits for all, with extra help for those most in need and no harm to those that need it the least. We consider this to be a strong indicator that larger studies with more generalizable results should be pursued. Other foci for future research would be the human factors and usability of the hardware and software interface to find the best motivational approach.

## Figures and Tables

**Table 1:** Questionnaire sent to staff members.

Question	Answer Choices
Do your co-workers’ habits with their sit–stand desk have an influence on the way you use your sit–stand desk?	-When we discuss our sitting/standing habits
	-When I see them using their sit–stand desk
	-Both of the above
	-No
In which way do they influence how often you stand using your desk?	-Motivates me to stand more
	-Makes me feel like I want to stand less
In which way do they influence how often you move your desk? (sitting to standing/standing to sitting)	-Motivates me to change positions more
	-Makes me feel like I want to change positions less

**Table 2:** Overall group means and pairwise comparisons.

	Baseline Mean (*SD*)	Intervention Mean (*SD*)	Pairwise Mean Difference (*SD*)	Paired Two Sample for Means *t*-Test *p*-Value
Active Computer Use (ACU) hrs	3.03 (1.11)	3.08 (1.15)	0.05 (0.42)	0.45
Sit-ACU hrs	2.32 (1.31)	2.21 (1.23)	−0.11 (0.72)	0.19
Stand-ACU hrs	0.71 (1.07)	0.86 (0.99)	0.15 (0.70)	0.12
Desk Position Changes Per Work Day	0.46 (0.72)	1.41 (1.36)	0.95 (1.20) *	<0.001 *
Max ACU hrs desk in a position before change	6.17 (1.79)	2.32 (0.61)	3.85 (0.65) *	<0.001 *

* Significant change (*p* < 0.001).
